# Revision and re-revision after mobile- and fixed-bearing lateral unicompartmental knee arthroplasty

**DOI:** 10.1007/s00264-026-06857-5

**Published:** 2026-05-21

**Authors:** Mustafa Canberk Göktepe, Jürgen Martin, Guido Mohr, Mustafa Hariri, Michael Clarius

**Affiliations:** 1 Department of Orthopaedics, University Hospital Heidelberg, Heidelberg, Germany; 2 Vulpius Hospital, Bad Rappenau, Germany

**Keywords:** Lateral unicompartmental knee arthroplasty, Fixed-bearing lateral UKA, Mobile-bearing lateral UKA, Revision, Re-revision, Total knee arthroplasty, Oxford UKA

## Abstract

**Background:**

Limited information is available about revision after lateral unicompartmental knee arthroplasty (UKA) due to infrequency of this procedure. The purpose of this study is to examine reason for failure, time to failure, patient characteristics, type of revision and re-revision surgeries after failed mobile- and fixed-bearing lateral UKA.

**Methods:**

In this single centre study, a total of 43 patients who underwent a revision surgery between 2010 and 2024 after mobile- and fixed-bearing lateral UKA implantation were analyzed. Demographic information, time to revision surgery, reason for failure, type of revision and re-revision surgery of these patients were evaluated.

**Results:**

In revision after mobile-bearing lateral UKA group with 20 patients, progression of osteoarthritis (OA) and bearing dislocation were the leading reasons for revision. In 13 cases, conversion to total knee arthroplasty (TKA), in four cases conversion to fixed-bearing component and in three cases bearing exchange were performed after a failed mobile-bearing lateral UKA. After revision, five patients underwent a re-revision surgery. In revision after fixed-bearing lateral UKA group with 23 patients, progression of OA was the leading reason for revision. In 20 cases, conversion to TKA, in two cases conversion to TKA with augments and in one case conversion to Vanguard 360 knee revision system with augments were performed. After revision to TKA, one patient underwent a re-revision surgery.

**Conclusions:**

TKA without augments could be used in 94% of cases, if revision to TKA is performed. Bearing exchange or revision to fixed-bearing lateral UKA resulted in a high re-revision rate of 57%, so alternative revision strategies should be considered after a failed mobile-bearing lateral UKA.

## Introduction

Unicompartmental knee arthroplasty (UKA) is an established surgical procedure for the treatment of anteromedial or lateral end-stage osteoarthritis (OA) [1]. According to arthroplasty registries, the incidence of UKA implantation has been increasing over the last decade significantly [2–5]. UKA offers a higher patient satisfaction rate and better patient-reported outcome measures (PROMs) in several areas compared to the Total Knee Arthroplasty (TKA) when treating unicompartmental OA [6, 7]. Furthermore, almost 50% of all patients with knee OA were described to be suitable for UKA implantation so that a further increase in UKA incidence might be expected in the near future [8].

Medial knee OA is almost ten times more frequent than lateral knee OA, hence there has been an extensive amount of research regarding medial UKA whereas there is still limited amount of data available about lateral UKA [9, 10]. Existing studies have shown high survivorship and good clinical outcomes at mid- and long-term follow-up after lateral UKA similar to medial UKA [11–20]. For lateral UKA, there have been two different bearing designs with mobile- and fixed-bearing. Recent clinical studies with medium and long-term follow-up have shown that mobile-bearing lateral UKAs have high revision rate due to bearing dislocation [21, 22]. Furthermore, revision after mobile- and fixed-bearing lateral UKA implantation has been a question of debate. There are only a few studies which examined revision surgeries after lateral UKA due to complexity and infrequency of this procedure. Most of these studies examined revision surgeries after failed mobile-bearing lateral UKA describing different revision strategies such as bearing exchange or conversion to a fixed-bearing tibial component in case of a bearing dislocation [21–23]. In case of progression of OA, component loosening or instability after mobile- or fixed-bearing lateral UKA, the existing studies, which have only a small sample size, show different revision strategies. In some studies, conversion to a TKA was the preferred revision method after a failed lateral UKA while conversion to a knee revision system was preferred in others [19–28]. Furthermore, information about the use, size and location of augments in revision surgeries after a failed lateral UKA is still lacking in the literature.

So far, there has yet to be a consensus on the right revision strategy after a failed mobile- and fixed-bearing lateral UKA due to limited number of studies and data. Therefore, the purpose of this study is to examine the reason for failure, time to failure, patient characteristics and more importantly type of revision and re-revision surgeries after a failed mobile- and fixed-bearing lateral UKA in a single centre multi-surgeon cohort.

## Materials and methods

In this retrospective study, patients who underwent a revision surgery between January 2010 and December 2024 after mobile- or fixed-bearing lateral UKA implantation were analyzed. Ethical approval was obtained from the institutional review boards of the University of Heidelberg (S-440/2025) and the study was conducted in accordance with the Helsinki Declaration of 1975. Demographic information such as gender, age at the time of revision surgery and body mass index (BMI) were recorded. Furthermore, important information such as type and size of primary lateral UKA implant, time between primary lateral UKA implantation and revision surgery, reason for failure, type of revision and re-revision surgery of these patients were analyzed. Demographic information, modes of failure, types of revision and re-revision surgeries were evaluated separately for mobile- and fixed-bearing lateral UKA groups.

A total of 841 lateral UKAs (193 mobile-bearing and 648 fixed-bearing lateral UKA) were implanted from January 2010 to December 2024 by two high volume surgeons in a single centre where the study was carried out. The indication for lateral UKA implantation was isolated lateral compartment OA with Kellgren-Lawrence classification grade IV. Preoperative standard radiological examination included anteroposterior (a.p.), lateral, varus-/valgus stress and Rosenberg views of the knee. The intactness of the anterior cruciate ligament, medial and lateral collateral ligaments were prerequisites for lateral UKA implantation which were evaluated radiologically and clinically. Furthermore, valgus deformity was manually correctable in all patients preoperatively. Fixed valgus deformity, flexion contracture more than 15 degrees and rheumatoid arthritis were contraindications for lateral UKA implantation. Lateral UKA implantation was performed using a lateral parapatellar approach with a cemented mobile-bearing Oxford domed lateral prosthesis (Zimmer Biomet Inc., Warsaw, Indiana, USA) or a fixed-bearing Oxford implant (Zimmer Biomet Inc., Warsaw, Indiana, USA) before 2016. After 2016, lateral UKA implantation was carried out using only a fixed-bearing Oxford implant. All revision surgeries were performed by the same two high volume surgeons (MC and JM). Conversion to TKA was performed using Zimmer Biomet Vanguard system.

A total of 43 patients who underwent a revision surgery between January 2010 and December 2024 after lateral UKA implantation were detected. 20 patients were implanted with a mobile-bearing lateral UKA whereas 23 patients were implanted with a fixed-bearing lateral UKA in index surgeries. Two of the implanted mobile-bearing Oxford implants were titanium niobium nitride coated due to metal hypersensitivity of two patients. In fixed-bearing lateral UKA group, one of the patients was implanted with a Sigma High Performance Partial Knee (DePuy Synthes, Raynham, USA) in an external clinic.

### Statistical analysis

Data was collected retrospectively using the clinic register. Clinical reports were compared with radiographic images to confirm the diagnosis and revision indication. Data collection and statistical analysis were performed using Excel software (Microsoft Corp, USA) and SPSS version 28.0 (IBM, USA). Mean age, BMI and time to revision were calculated. Mean values were displayed as the mean ± standard deviation (SD). Group comparisons were performed using independent-samples t-test. The *p-*value was considered statistically significant when *p* < 0.05. The relationship between reason for failure and time from index arthroplasty to revision surgery was depicted using boxplots. Modes of failure and type of revision surgeries after failed mobile- and fixed-bearing lateral UKA were summarized in pie and flow charts.

## Results

In mobile-bearing lateral UKA group, 14 women and six men underwent a revision surgery. This group had a revision rate of 10%, considering a total of 193 index surgeries with mobile-bearing lateral UKA were performed during the study period. Mean age at the time of revision surgery was 64 ± nine years while mean BMI was 29 ± 4 kg/m^2^. Mean time from index arthroplasty to revision surgery was 4,6 ± 4,2 years (Table 1).

**Table 1:** Demographic data for patients undergoing a revision surgery after mobile-bearing lateral UKA

Patient Demographics	
Number of patients	20
Women/Men	14/6
Right/Left	13/7
Age	64 ± 9
BMI (kg/m^2^)	29 ± 4
Time to revision surgery (years)	4,6 ± 4,2

In fixed-bearing lateral UKA group, 19 women and four men underwent a revision surgery. This group had a revision rate of 3%, considering a total of 648 index surgeries with fixed-bearing lateral UKA were performed during the study period. Mean age at the time of revision surgery was 66 ± 12 years and mean BMI was 29 ± 4 kg/m^2^. Mean time from index arthroplasty to revision surgery was 2,1 ± 2,0 years (Table 2).

**Table 2:** Demographic data for patients undergoing a revision surgery after fixed-bearing lateral UKA

Patient Demographics	
Number of patients	23
Women/Men	19/4
Right/Left	11/12
Age	66 ± 12
BMI (kg/m^2^)	29 ± 4
Time to revision surgery (years)	2,1 ± 2,0

No statistically significant difference was observed in age (*p* = 0.43) sex (*p* = 0.34) and BMI (*p* = 0.92) between mobile- and fixed-bearing lateral UKA groups.

### Revision and re-revision after mobile-bearing lateral UKA

**Fig. 1:**
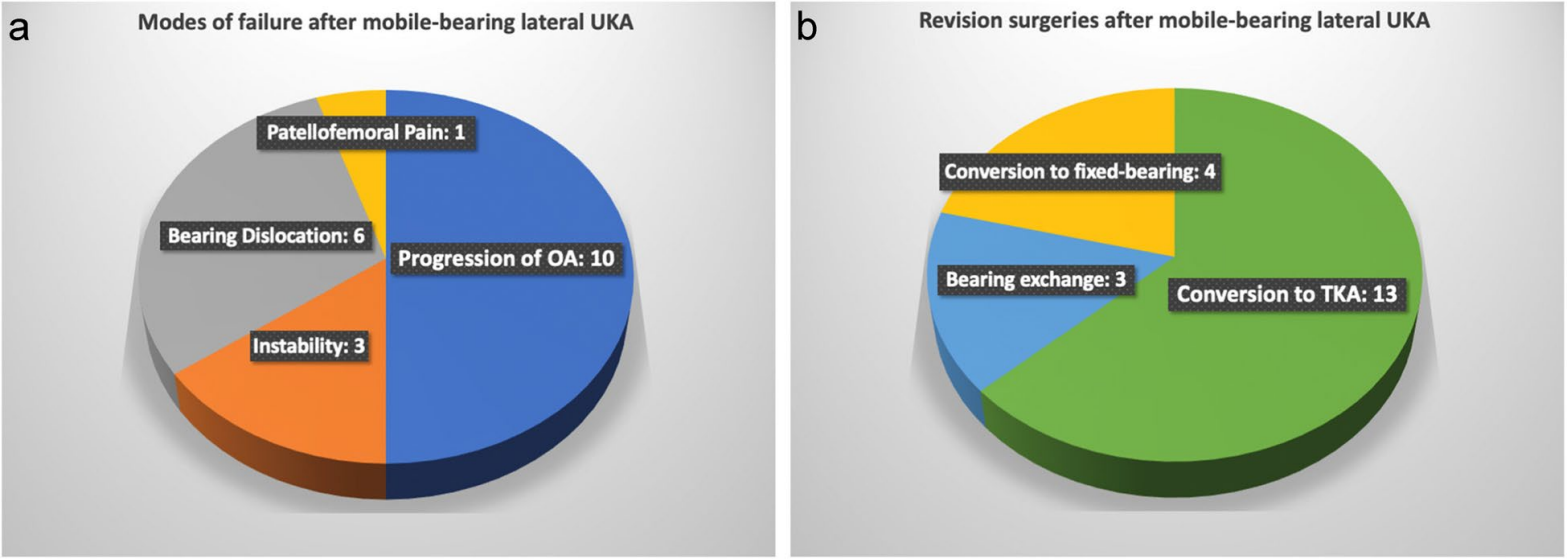
**a** Pie chart depicting modes of failure after mobile-bearing lateral UKA implantation, OA: osteoarthritis (**b**) Pie chart depicting type of revision surgeries after failed mobile-bearing lateral UKA implantation, TKA: total knee arthroplasty

Progression of OA in medial compartment was the leading reason for failure after mobile-bearing lateral UKA implantation with ten cases. In eight of these cases, progression of OA occurred in medial compartment whereas in one case progression of OA in retropatellar compartment was the reason for failure. Furthermore, in another case simultaneous progression of OA in medial and retropatellar compartment was observed. The other causes of failure and revision after mobile-bearing lateral UKA were bearing dislocation in six cases, instability in three cases and patellofemoral pain in one case (Fig. 1). No revision surgery was necessary for a periprosthetic infection.

**Fig. 2:**
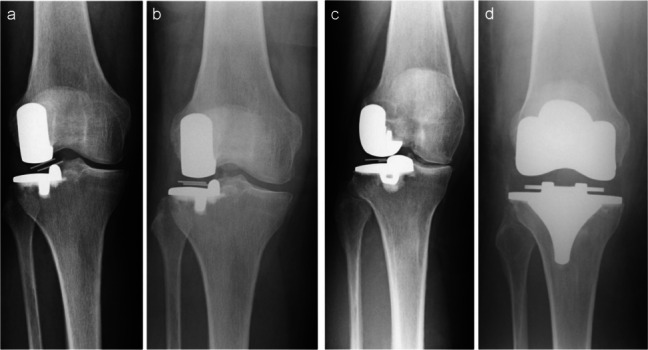
**a** A. p. radiograph demonstrating a bearing dislocation after mobile-bearing lateral UKA (**b**) Postoperative a. p. radiograph after revision surgery with bearing exchange (**c**) A. p. radiograph demonstrating progression of OA in medial compartment after mobile-bearing lateral UKA (**d**) Postoperative a. p. radiograph after conversion to TKA

In 13 cases, a conversion to TKA was performed without any additional augments. In four cases with recurrent bearing dislocation, tibial component was converted to a fixed-bearing component while a bearing exchange with a higher mobile-bearing was performed in three cases after bearing dislocation with lateral instability (Figs. 1 and 2).

**Fig. 3:**
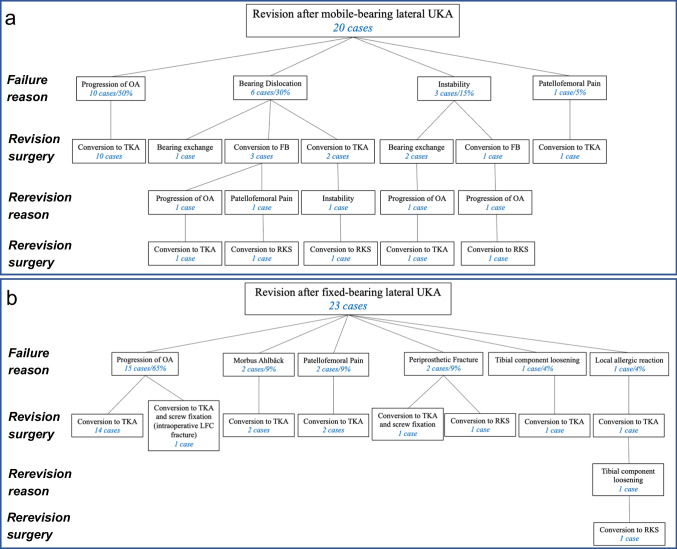
Flow chart depicting modes of failure and revision strategies after mobile- (**a**) and fixed-bearing (**b**) lateral UKA implantation, TKA: total knee arthroplasty, RKS: revision knee system, LFC: lateral femoral condyle

After the first revision surgery, five patients presented with complaints once again. A patient with a revision to TKA from mobile-bearing UKA after recurring bearing dislocation had complaints due to instability three years after the first revision surgery. A re-revision surgery and conversion to EnduRo Rotating Hinge Knee System (Aesculap AG, Tuttlingen, Germany) with femoral (8 mm, lateral and medial) and tibial augments (8 mm, lateral and medial) was performed. Another patient had an anterior knee pain 14 years after a revision to a fixed-bearing tibial component which was treated with a re-revision to femoral primary TKA component and to a Vanguard 360 tibial component with augment (10 mm, lateral and medial) due to a tibial osseus defect. In the case of lateral instability with mobile bearing UKA, a revision to TKA was performed four years after a bearing exchange. In a similar case, a re-revision to TKA was carried out due to progression of arthritis in retropatellar compartment six years after revision to fixed-bearing tibial component. One patient was re-revised to TKA with a tibial augment (5 mm, lateral) due to a progression of OA in medial compartment 11 years after revision to a fixed-bearing tibial component (Fig. 3).

### Revision and re-revision after fixed-bearing lateral UKA

**Fig. 4:**
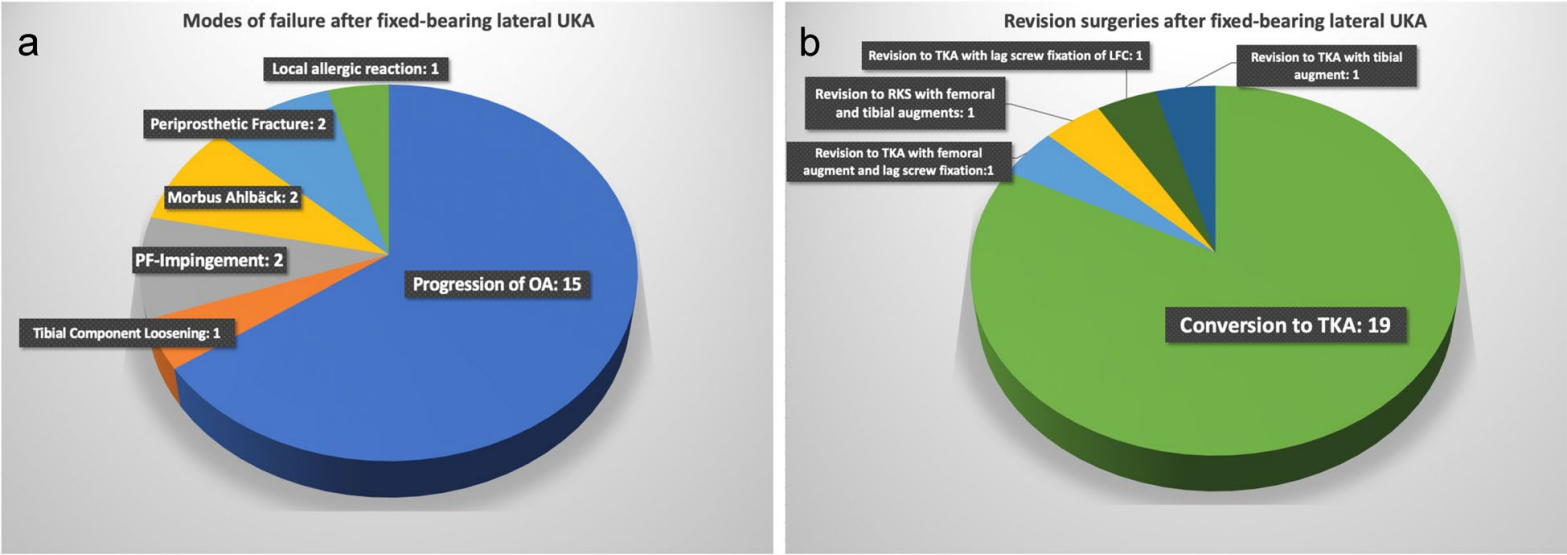
**a** Pie chart depicting modes of failure after fixed-bearing lateral UKA implantation, OA: osteoarthritis, PF-Impingement: patellofemoral impingement (**b**) Pie chart depicting type of revision surgeries after fixed-bearing lateral UKA implantation, TKA: total knee arthroplasty, RKS: Revision Knee System, LFC: lateral femoral condyle

In 15 cases, progression of OA was the leading reason for failure after fixed-bearing lateral UKA implantation. The other causes of failure and revision was patellofemoral impingement in two cases, Morbus Ahlbäck in two cases, a periprosthetic femur fracture, a periprosthetic lateral tibia fracture, a loosening of tibial component after polyethylene (PE) bearing wear and a local postoperative allergic reaction (Fig. 4). No revision surgery was necessary for a periprosthetic infection.

**Fig. 5:**
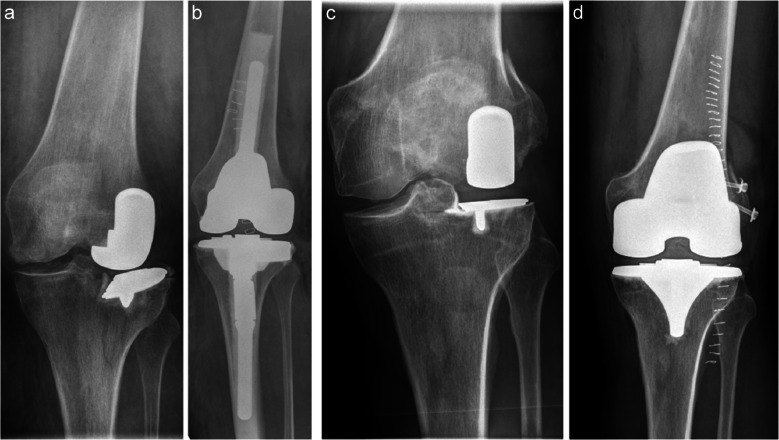
**a** A. p. radiograph showing a periprosthetic lateral tibia fracture after fixed-bearing lateral UKA (**b**) Postoperative a. p. radiograph after revision surgery with Vanguard 360 knee revision system with femoral (10 mm, lateral) and tibial (10 mm, lateral) augments (**c**) A. p. radiograph demonstrating a periprosthetic femoral fracture after fixed-bearing lateral UKA (**d**) Postoperative a. p. radiograph after revision to TKA and lag screw fixation of lateral femoral condyle

In 19 cases, a conversion to TKA was performed without any additional augments. In only one case after progression of OA, a tibial augment (5 mm, posterior lateral) was needed during revision to TKA. In the case of a periprosthetic lateral tibia plateau fracture, the lateral UKA was converted to the Vanguard 360 knee revision system with femoral (10 mm, lateral) and tibial (10 mm, lateral) augments due to a large femoral and tibial osseous defect (Fig. 4). In one case, an intraoperative lateral femoral condyle fracture occurred during revision to TKA which was fixed with a lag screw. Furthermore, a posteromedial augment (5 mm) was used due to a tibial and posteromedial femoral osseous defect. In the case of another periprosthetic femoral fracture, a revision to TKA in addition to a lag screw fixation was performed (Figs. 4 and 5).

One patient after revision to a hypoallergenic coated TKA due to an allergic reaction had a loosening of the tibial component after two years. A two-stage revision surgery was performed due to a suspected infection. After TKA removal and spacer implantation, a re-revision to Vanguard 360 knee revision system with femoral augments was performed (Fig. 3).

## Discussion

In this single-centre multi surgeon cohort, the most common reason for failure was the progression of OA, followed by bearing dislocation, patellofemoral impingement, instability, periprosthetic fracture, osteochondrosis dissecans, tibial component loosening, local postoperative allergic reaction and postoperative pain with limited range of motion. Conversion to TKA was the most common revision choice after a failed lateral UKA.

**Fig. 6:**
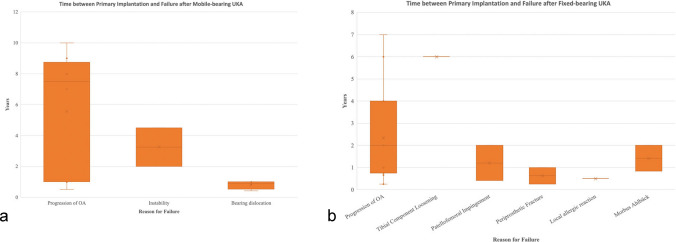
Boxplot depicting the time between primary implantation and reason for failure after mobile-bearing (**a**) and fixed-bearing (**b**) lateral UKA implantation

In the literature, there are only a few studies about modes of failure and revision surgeries after lateral UKA implantation. So far, the existing studies have a limited number of cases while some studies did not describe the type of primary bearing component [24, 28]. Moreover, most of the studies reported on revision cases after lateral UKA with mobile-bearing component [21, 23]. To authors’ knowledge, the case series by Citak et al. is one of the few studies which focused on the modes of failure and revision of failed lateral UKAs as 16 patients with failed lateral UKAs were reviewed. While progression of OA was the most common reason for failure similar to this study. Revision knee systems were used in 13 cases whereas only three cases were converted to TKA in contrast. Another difference between our study and the study by Citak et al. is the mean time to failure as time to failure after lateral UKA implantation was longer in the study of Citak et al. (Fig. 6). The ratio of the cases with polyethylene wear which is a reason for a late implant failure was higher in the cohort of that study. This might have caused the difference in time to failure. On the other hand, important details such as type of lateral UKA implant and bearing or if there were any re-revision surgeries were missing so that a comparison of these important parameters was not possible [24].

There are other studies which analyzed mid- to long-term results after lateral UKA implantation. The study by Kennedy et al. presented the results of Oxford mobile-bearing domed lateral UKA describing 34 revisions in 325 UKAs in mid- to long term follow-up. Bearing dislocation was the most common reason for failure in that study, followed by progression of OA [21]. Lustig et al. examined 49 fixed-bearing lateral UKAs after five to 16-year follow-up and described only four revision surgeries. Three out of four revision surgeries were due to progression of OA [16]. In a multicenter study, Deroche et al. analyzed 268 fixed-bearing lateral UKAs from five different implant models at five to 23 years’ follow-up. In that study, only eight revision surgeries were described and progression of OA was the leading cause for revision surgeries followed by tibial loosening [19]. In a single surgeon cohort study by Harkin et al., only 11 revision surgeries were described after lateral UKA implantation where progression of OA was the leading cause [28]. A study by Burger et al. which analyzed Dutch Arthroplasty Register also showed that progression of OA was the most common reason for failure after mobile- and fixed-bearing lateral UKA [26]. In most of these studies, revision to TKA was the most common treatment choice after lateral UKA. However, detailed information whether augments were used in conversion to TKA was missing.

This study addresses the limitations of previous studies, which have a small sample size, by presenting a total of 43 revision cases after lateral UKA. Furthermore, the type of implants, size and position of augments used in revision surgeries were described in detail. In this study, progression of OA was the leading cause for revision surgeries after mobile- and fixed-bearing lateral UKA implantation. In revision surgeries after mobile- and fixed-bearing lateral UKA, augments were only used in three instances. In a revision surgery after progression of OA, a 5 mm posterolateral tibial augment was needed. In the other two cases, an osseous defect caused by a periprosthetic fracture was the reason for using augments.

On the other hand, this study shows that not only progression of arthritis but also bearing dislocation could be a major risk for revision and re-revision after mobile-bearing lateral UKA. Five out of six patients with re-revision surgeries were primarily operated with a mobile-bearing lateral UKA. This finding also suggests that fixed-bearing implants could have an advantage over mobile-bearing components in primary lateral UKA regarding re-revision surgeries.

In mobile-bearing lateral UKA group, four revision surgeries were performed after bearing dislocation with conversion to fixed-bearing component, three of which were re-revised due to progression of OA in two cases and patellofemoral pain in one case. Thus, conversion to fixed-bearing component after failed mobile-bearing lateral UKA had a very high re-revision rate with 75%. Furthermore, three revision surgeries were performed with bearing exchange after bearing dislocation, one of which was re-revised due to progression of OA. According to these findings, conversion to TKA might be a better revision strategy instead of revising to a fixed-bearing component after failed mobile-bearing UKA with recurrent bearing dislocation. However, it should also be noted that there are some contradicting findings in the literature. For example, Hariri et al. described a 100% survival rate in three cases after exchange of the tibial component to a fixed-bearing design whereas the survival rate after bearing exchange was 0% in six cases after a bearing dislocation. The authors concluded that conversion to a fixed-bearing tibial component is a suitable revision strategy addressing recurrent bearing dislocation in contrast to the findings of this study [23].

This study has certain limitations. The inherent bias resulting from the retrospective study design is one of the most notable limitations of this study. Another important limitation is that PROMs and their correlation with different revision strategies are missing. This limits not only clinical assessment of different revision strategies but also interpretation of revision success in terms of patient satisfaction and functional status which are important to facilitate informed decision-making in case of a failed lateral UKA. However, the primary objective of this study was to examine the modes of failure, time to failure, application of augments, type of revision and re-revision surgeries after a failed lateral UKA since there is still a substantial knowledge gap in the literature. To authors’ knowledge, this is the largest single-center study describing revision and re-revision surgeries after mobile- and fixed-bearing lateral UKA, thus providing an important contribution to the literature.

This study suggests that conversion to TKA can be a reliable treatment option after failed mobile- and fixed-bearing lateral UKA especially when progression of OA occurs. In 94% of our patients regular TKA could be performed without any augments if revision to TKA is performed. Revision with augments and knee revision system was rather an exception being used only in 6% of our patients in this study cohort. Furthermore, even with femoral or tibial osseous defect, conversion to TKA could be performed with the addition of augments before converting to a knee revision system except one case with periprosthetic proximal tibia fracture. Future studies could further clarify the clinical relevance of different revision strategies incorporating PROMs.

## Conclusion

In this study, progression of OA was the most common cause of revision after mobile- and fixed-bearing lateral UKA. If revision to TKA is performed, TKA without augments could be used in 94% of the study population. Bearing exchange or revision to a fixed-bearing tibial component design resulted in a high re-revision rate, so alternative revision strategies should be considered after a failed mobile-bearing lateral UKA.

## Data Availability

No datasets were generated or analysed during the current study.
